# Effects of Intermittent Aerobic Training on Passive Avoidance Test (Shuttle Box) and Stress Markers in the Dorsal Hippocampus Of Wistar Rats Exposed to Administration of Homocysteine

**Published:** 2013

**Authors:** Somayeh Hosseinzadeh, Valiollah Dabidi Roshan, Mehdi Pourasghar

**Affiliations:** 1 Babol University of Medical Sciences, Babol, Iran.; 2 Asociate professor, College of Physical Education and Sport Sciences, department of sport physiology, University of Mazandaran, Babolsar, Iran.; 3 Psychiatry and Behavioral Sciences Research Center, Department of Psychiatry , Mazandaran University of Medical Sciences , Sari, Iran.

**Keywords:** Attitude, Consultation-Liaison Psychiatry, Practice

## Abstract

**Objective**: Elevated amino acid homocysteine (Hcy) levels and insufficient physical activity are the risk factors in Alzheimer disease (AD) development. The effect of intermittent aerobic training on memory retention test and Thiobarbituric Acid Reactive Substances (TBARS) and superoxide dismutase (SOD) levels in the dorsal hippocampus of rats which were stimulated with Hcy is investigated.

**Methods:** In order to determine the dose at which using Shuttle Box Test recognizes degenerative changes and/or memory impairment, 40 rats were injected by different dosages of Hcy to the dorsal hippocampus. It was observed that the required Hcy dose is 0.6 M. Then 44 rats were divided into four groups including training and control groups at 4 weeks of aerobic exercise in training and control groups at 8 weeks. To determine the effect of homocysteine on the memory impairment, Shuttle Box Test was used on treadmill (5 sessions/week, 12-18 m/min and 10-58.5 min).

**Results:** Hcy administration caused memory impairment and significant increase in TBARS. Significant decrease in TBARS level was noted after 8 weeks of aerobic exercise, but not after just 4 weeks of exercise compared with control group. In addition, performing 8 weeks of aerobic training led to significantly increased superoxide dismutase (SOD) level and the time of avoidance learning test.

**Conclusion:** Hyperhomocysteinemia caused learning and memory deficits probably by generating reactive oxygen species (ROS) and the present study showed that regular moderate intensity intermittent exercise may reverse this process and exercise is recommended as a strategy to improve symptoms of senile neurodegenerative disease .

**Declaration of Interest:** None.

## Introduction

Alzheimer's disease (AD), the most prevalent form of dementia, is a multifactorial neurodegenerative dysfunction which is characterized by memory impairment and progressive decline of cognitive functions (1). Its prevalence has been increasing as a consequence of the increase in life expectancy in the modern society (1,2) and significantly impacts patients, families, caregivers, communities and society as a whole (3). Neuropathological manifestations of AD include formation of amyloid-β (Aβ) peptide plaques and neurofibrillary tangles (1,2). The cause of neuronal death in AD in key parts of the brain, such as the hippocampus, is not fully known yet. But elevated serum amino acid homocysteine (Hcy) and insufficient physical activity are discussed as the potential risk factors in this disease (4). Further support for an association between total Hcy (tHcy) and dementia comes from studies showing associations between high tHcy and lower cognitive function among non-demented individuals as well as with increased conversion rate from mild cognitive impairment to AD (5). An increment in the plasma Hcy level of 5 μmol per liter increased the risk of AD disease by 40 percent (6). The magnitude of the association between Hcy and AD may increase with age and appears to plateau around age 60 (3).

Hcy is a sulphydryl-containing amino acid (1), derived from the essential amino acid methionine. Its metabolism depends on the B vitamins cobalamin (vitamin B12), pyridoxine (vitamin B6) and folic acid (1,2). The mechanism by which high Hcy levels causes its neurotoxic effects is not fully understood. Potentiation of Aβ peptide neurotoxicity by homocysteine has been described (2). Recently, it has been suggested that chronic administration of Hcy to the rats affected both long- and short-term memories and it was purposed that hyperhomocysteinemia causes memory impairment by enhancing oxidative stress (7). Oxidative stress is a relative increase in the ratio of free radicals to antioxidants (3).

It is known that Hcy reduces levels of all major antioxidants in patients with cognitive impairments or AD (8). Moreover, Hcy has ability to inhibit the expression of antioxidant enzymes such as glutathione peroxidase (GSH-Px) and superoxide dismutase (SOD) which might potentiate the toxic effects of reactive oxygen species (ROS) (7).

Persons with cognitive disorders as a consequence of cognitive decline often need long-term care that leads to institutionalization. Nursing homes and most institutional settings generally lack proper environmental stimulation and physical activity opportunities (9). Some factors, like environmental enrichment and exercise, may promote functional and behavioral recovery and solve these problems partly. 

It has been generally accepted that exercise produces benefits for overall health (10), In recent years, there has been a strong interest in physical activity as a primary behavioral prevention strategy against cognitive decline, especially in elderly people (10,11). However, there are studies showing no effect of exercise, and even a few describing negative effects of exercise (12). It seems that the kind, duration, and intensity of physical activity is important (13,14). Most prospective intervention studies of exercise and cognition to date have focused on aerobic-based exercise training. These studies highlight that aerobic-based exercise training enhances both brain structure and function. However, it has been suggested that other types of exercise training, such as resistance training, may also benefit cognition. Resistance training may prevent cognitive decline among seniors via mechanisms involving insulin-like growth factor I and Hcy(11). Kim et al. (9) investigated the effect of treadmill exercise on memory in relation to neurogenesis and apoptosis in the hippocampal dentate gyrus of old-aged rats (15,16). Microarray analysis has shown that both acute and chronic voluntary exercise affect the expression of hippocampal genes related to synaptic plasticity. In particular, genes related to the glutamatergic system are up-regulated, whereas those related to the gamma-aminobutyric acid (GABA) system are down-regulated. Glutamatergic function in the dentate gyrus may influence the production and function of new neurons in the adult brain (16). Physical activity leads to enhance NO release which may generate improved cerebrovascular function, enhanced anti-inflammatory activity, and may lead to increased acetylcholine release (14).

Despite the growing evidence on the benefits of exercise for the health and functioning of older adults with cognitive disorders, the available literature lacks clinical evidence that supports recommendations for exercise guidelines and testing in older persons with dementia and related cognitive impairments (9). Based on this fact that elderly persons, specially Alzheimer's patient cannot do exercise continuously and for long-term periods, in this study, we investigated the effect of 4 and 8 weeks of intermittent aerobic training on memory retention test and thiobarbituric acid reactive substances (TBARS), the most abundant product arising from lipid peroxidation, and SOD levels in the dorsal hippocampus of rats which stimulated with Hcy.

## Materials and Methods

All experiments were performed in accordance with the guidelines provided by the Experimental Animal Laboratory (15). The study protocol was approved by the Department of Physiology, Mazandaran University and was performed according to the guiding procedures in the Care and Use of Animals, prepared by the Council of the American Physiological Society (15).

Eighty-four Wistar rats (3 months of age and mean weight of 300±40g) were randomly assigned into two groups after familiarization to the lab environment: 

1) The pilot study protocol (40 rats): For this group, injection of Hcy into the dorsal hippocampus was done at doses of 0.1 (n = 10), 0.2 (n = 10), 0.3 (n = 10) and 0.6 (n = 10) molar in order to recognize at which dose using shuttle box test degenerative changes and/or memory impairment occurs. For this purpose, these rats were transmitted to the research site and kept there for 3 weeks and then after surgery and cannula investment in the dorsal hippocampus, they had 1 week respite to recovery. The different doses of Hcy (0.1, 0.2, 0.3 and 0.6 molar) were injected through this cannula by Hamilton Syringe and all groups were examined with passive avoidance retention test (shuttle box). Finally, it revealed that rats developed memory impairment at doses of 0.6 molar of Hcy. So that, 0.6 molar Hcy was injected to the rats in “main study” group as the effective dose. 

2) The main study protocol (44 rats): They were randomly divided into four groups including: training and control groups at 4 weeks and training and control groups at 8 weeks. 

Rats in main groups underwent surgical intervention like pilot groups. Then, Hcy 0.06 molar was administered at dorsal hippocampus via cannula. Afterwards, they performed training protocol for 4 weeks and 8 weeks and finally brain removed and hippocampus tissue seperated in 3 process (base, 4 and 8 week) and then samples were analyzed.

*Passive avoidance test (PAT)(shuttle box)* *:*

The apparatus (shuttle box) consisted of equal sized light and dark compartments (20 × 80 × 20 cm). The apparatus consisted of two-compartment dark/light shuttle box. The two compartments were separated by a guillotine door (7 × 9 cm) that could be raised to 10 cm. Floor of the dark compartment consisted of a stainless steel shock grid floor. Electric shocks were delivered to the grid floor with a stimulator (50 Hz, 1.5 sec, 1.5-2 mA intensity). This test has two stages, instruction and memory test. In stage one, each rat was placed in the larger illuminated compartment and after 5 seconds, the door was opened and the rats were allowed to move freely into the dark chamber. Upon entry into the dark chamber, the door was closed and the rat was given 1 mA electrical shock in 3 seconds. After 20 seconds, the rat was returned to its home cage. Two minutes later, the rat was again placed in the larger illuminated compartment and the delay in entering the dark compartment was recorded. If the rats did not enter the dark compartment during 120 seconds, successful acquisition of passive avoidance response would be recorded (7,12), but in stage 2, which was performed 24 hours after stage one, each rat was re-tested in the same way, without stimulation, based on instruction in stage one and the delay in entering the dark compartment was recorded to a maximum of 300seconds. Short latencies indicate poor retention compared to significant longer latencies.

*Intracerebroventricular (i.c.v.) injection* *:*

The rats were anaesthetized with ketamin/xylazine (54 mg/kg) and placed in a stereotaxic frame. The rat skull was orientated according to Paxinos and Watson stereotaxic atlas. After a sagittal incision, the bregma suture was located and holes were drilled with an electrical drill at the following co-ordinates; 0.8 mm posterior to bregma, 1.5 mm lateral to the sagittal suture, and 3.7 mm ventrally. Care was taken not to damage the meninges. A Hamilton syringe with a cannula of diameter of 0.3 mm was used to inject 1 μl of solutions of Hcy (0.1, 0.2, 0.4 and 0.6 Molar) or Phosphate Buffered Saline (PBS) solution. The injection was carried out in the brain ventricle at a rate of 1 μl per 2 minutes. The cannula was left in situ for a further 5 min following Hcy injection to allow passive diffusion from the cannula tip and to minimize spread into the injection tract. The cannula was then slowly removed from the scalp, which was then closed with sutures (15).

*Hcy preparation and administration* *:*

Hcy powder was dissolved in hydrochloric acid (1Molar) and diluted with PBS (12). The pH of the solution was regulated at 7.4 by addition of 0.1 N NaOH. Solutions of Hcy were prepared freshly at concentration of 0.1, 0.2, 0.3 and 0.6 Molar (12). In the control group, Hcy solvent (PBS) and in the other groups, Hcy (0.6 μmol/μl) was injected (i.c.v)

*Intermittent aerobic training* *:*

At first, rats were familiarized with laboratory environment and running on rodent treadmill with speed of 5-8 m/min in 5-10 min with no gradient. After 2 weeks, training animals performed the intermittent running exercise of 12 to 18 m/min for 10 to 58.5 min, 5 times a week for 8 weeks without slope. Training speed in 2 initial weeks was steady because of surgery and physiological adaptations. Also, resting time/training ratio was considered at 1/4.

*Tissue preparation and SOD and TBARS analysis* *:*

All groups were anesthetized with ketamine and xylazine, sacrificed and then decapitated. The brain was removed carefully and the two hemispheres were separated along the midline. The hippocampus from each hemisphere of the brain was micro-dissected and homogenized in PBS solution (0.1 Molar) immediately and stored at -70° C for use in biochemical analysis. SOD (U/mg protein) and TBARS (nmol/g protein) levels in the dorsal hippocampus were measured by using ELISA and spectrophotometry, respectively. 

*Statistical analyses:*

Statistical analysis was performed using a commercial software package (SPSS version 16.0 for Windows). Results are expressed as means ± standard deviation. A one-way ANOVA test was used to detect statistical difference between the groups. Furthermore, the Tukey test was performed to establish changes differences in the mentioned markers between groups. The differences were considered significant at p< 0.05. 

## Results

**Figure 1:**
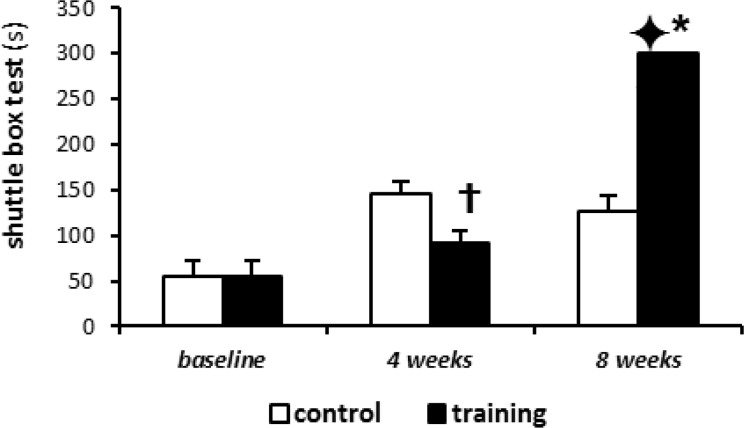
Shuttle box test results presented as mean (±SD)

Administration of 0.6 molar of Hcy into the dorsal hippocampus caused memory impairment (P< 0.001) (Figure 1).

*Significant difference compared to 4-week aerobic exercise

Significantly different from baseline.

**Figure 2:**
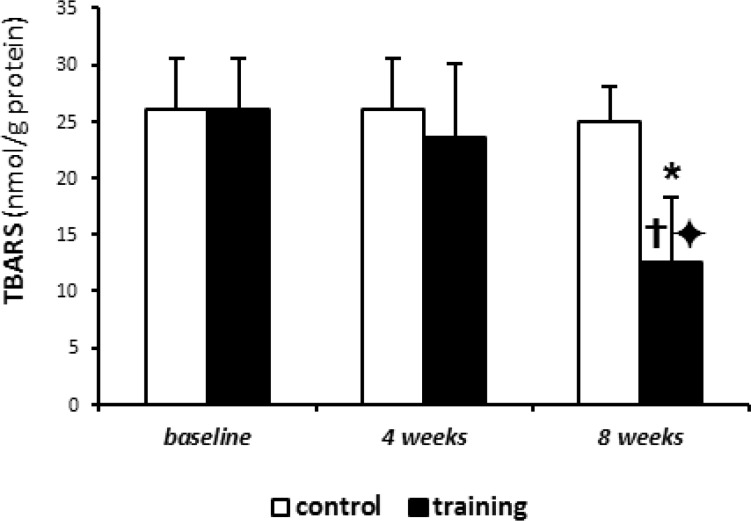
Thiobarbituric acid reactive substance (TBARS) levels. Results presented as mean (±SD)

**Figure 3:**
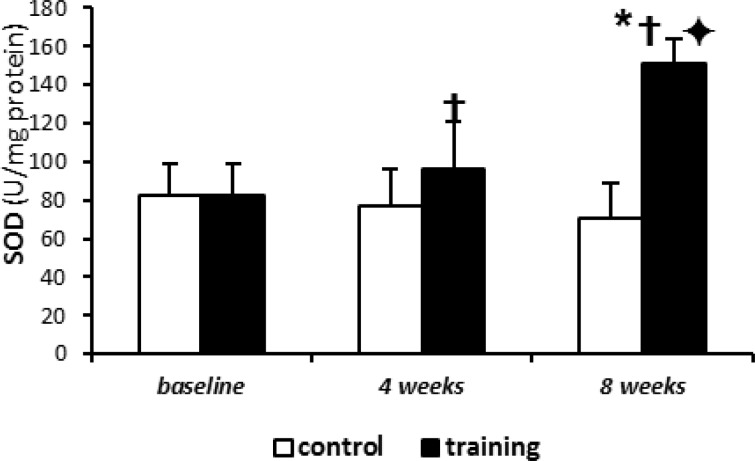
Superoxide dismutase (SOD) concentrations. Results presented as mean (±SD)

Significant difference compared to control group

also increased significantly TBARS concentration (Figure 2) (p< 0.001), and decreased SOD levels (p< 0.001) (Figure 3). On the other hand, the 4-week aerobic intermittent exercise decreased TBARS, as compared with 4-week control group (P= 0.391) (23.6±6.5 against 26.1±4.5 in 4-week control group); (Figure 2).

*Significant difference compared to 4-week aerobic exercise

Significantly different from baseline

Significant difference compared to control group

This TBARS decrease was statistically significant after 8-week exercise (P= 0.004) (12.6±5.8 against 25±.3.1 in 8-week control group). Also, SOD levels significantly increased after intermittent training compared with control group (Figure 3), although these changes were more elucidated after 8 weeks of training rather than 4 weeks (96.4±24.6 in 4 weeks training group against 76.6±19.6 in 4 weeks control group and 151.5±12.5 in 8 weeks training group against 70.5±18.6 in 8 week control group) (P values obtained for SOD levels in 4 weeks and 8 weeks training groups were 0.002 and 0.033, respectively). In addition, the 4 and/or 8 weeks of aerobic intermittent exercise led to increased time of avoidance learning test (Shuttle Box), as compared with baseline (92.6±12.8 in 4 weeks training and 300±1.0 in 8 weeks training against 54.2±18.4 in base group). So that, the increased time of avoidance test in 8 weeks training group was significantly different compared to 4 weeks training group (Figure 1).

*Significant difference compared to 4-week aerobic exercise

Significantly different from baseline;

Significant difference compared to control group

## Discussion

Hyperhomocysteinemia can cause brain damage both directly, through neuronal and vascular effects, and indirectly, through folate and Vitamin B12 deficit (17). In vivo studies with chronic subcutaneous Hcy injections in newborn rats suggested that Hcy can induce memory impairments in the water maze task (18). Chronic hyperhomocysteinemia impaired performance of rats in a water maze task and the retention of long-term memory in a passive avoidance task (7). The results of this study showed that the administration of 0.6 M Hcy into the dorsal hippocampus caused memory impairment. Thus, we suggest here that 0.6 μmol/μl of Hcy could impair the memory significantly, and decrease latency time. The effects of Hcy on the memory have been investigated in some of the earlier studies. Miller reported that hyperhomocysteinemia is associated with poor performance on objective tests of cognitive function in both case-control studies of patients with AD and cross-sectional, population-based studies of community-dwelling elderly people (19). It has been found that long-term exposure to Hcy can induce alteration in spatial learning, hippocampal signaling and synaptic plasticity (15,20). Although several mediating or modifying mechanisms that may underlie the negative effect of elevated tHcy (total Hcy) on cognitive functioning and the central nervous system have been implied, still no definitive answers exist. One potentially important mechanism that is increasingly linked to dementia and cognitive decline is systemic inflammation. 

Hyperhomocysteinemia has been associated with increased inflammation. A synergistic effect of both factors could be hypothesized, leading to faster cognitive decline and dementia (21).They found that high serum C-reactive protein (CRP) levels, especially in association with concurrent high serum Interleukin-6 (IL- 6) levels was a significant predictor of the risk of vascular dementia independently of hyperhomocysteinemia(21). Hcy may also be directly associated with pathogenic mechanisms of AD, such as stimulation of beta-amyloid production, down regulation of cholinesterases, activation of glutamate receptors, induction of tau phosphorylation , upregulation of presenilin 1, BACE and amyloid-beta , and oxidative stress (5). Zylberstein et al. expressed that higher tHcyin midlife is related to increased risk of AD in old age (5).

In agreement with the present results in which we demonstrated that Hcy can increase oxidative stress possibly through increasing TBARS and inhibition of SOD, previous studies suggested that Hcy might generate reactive oxygen species (ROS), which attack the poly unsaturated fatty acids (PUFA) of neuronal cell membranes and induce lipid peroxidation in the rat brain (15). Moreover, Hcy is able to prevent direct inactivation of NO by superoxide radicals generated during its auto-oxidation which lead to formation of peroxynitrite radicals that may cause further oxidative injury (7). Brain tissue is especially vulnerable to oxidative attack due to its relatively low antioxidant capacity, high consumption of oxygen, high content of polyunsaturated fatty acids, and high content of redox-active transition metals such as iron (3). In vitro studies using neuronal cell cultures suggest that Hcy may induce apoptosis and affect general metabolic activity (18).

Over the past several decades, increasing attention has been paid to the identification of interventions such as lifestyle habits which could be efficient in prevention of cognitive decline and dementia in elderly persons (22). The potential of physical activity to enhance cognition has been previously well‐supported by numerous studies (23-25). The results of the present study showed that regular physical activity restore the damage caused by administration of Hcy and improve memory retention and cognitive function in the brain through modification of oxidation/reduction homeostasis. The interest in hippocampal-dependent tasks derives from the fact that exercise increases angiogenesis, neurogenesis and synaptic plasticity (10), and induces nervous protection through enhancing activation of endogenous antioxidant systems in this structure (26).In accordance with previous findings in which suggested that with respect to animal studies on learning and memory, physical exercise has been shown to have a beneficial effect on water maze, passive avoidance, pole-jumping active avoidance, fear conditioning to the context and radial arm maz(10), we showed that 4 and 8 weeks aerobic training had positive effects on redox homeostasis and memory retention test by shuttle box, but long-term physical activity is associated with better improvements in rodents. 

Cross-sectional and longitudinal data have demonstrated that physically active people have a lower risk of developing disease and related cognitive disorders when compared with sedentary people. Most studies focused on the short-term effects of exercise and physical activity. Frail sedentary older adults have good short-term responses to physical exercise. There is a need for long-term follow-up and longitudinal investigations with this population. The results of a meta-analysis suggest a medium to large treatment effect for health-related physical fitness components, and an overall medium treatment effect for combined physical, cognitive, functional, and behavioral outcomes (9). Exercise is known to be one of the most potent vasoprotective non-pharmacological interventions. Its value in primary and secondary AD prevention may thus be twofold: first, exercise may directly counteract vascular oxidative stress specifically found in AD (e.g., by antagonizing ROS production and by improving AD-typical cerebral blood flow reduction). Second, it may reduce AD risk indirectly by reducing the vascular burden as a critical factor for its onset. This could be achieved by its additional beneficial effects on vascular NO metabolism, vascular remodeling/regeneration and endothelial signaling (27). Similarly, Heyn et al. provided preliminary evidence for the effectiveness of exercise treatments for persons with dementia and related cognitive impairments (9).It is now well established that cognitive improvement and neurogenesis following exercise critically depend on synthesis and release of Brain Derived Neurotherophic Factor (BDNF), most likely of endothelial origin and regulated by vascular endothelial growth factor (VEGF) (27). Podewils et al. showed that a higher amount of estimated energy expenditure and higher diversity of physical activity was associated with a lower incidence of both Alzheimer and vascular dementia (28).In part, this is probably due to the fact that, together with positive results, there are also many studies showing no effect of exercise, and even a few describing negative effects (12). This aspect, the type of exercise, seems to be very important in determining the effect of the treatment over different neurochemical variables (10). Garcia-Capdevila et al. showed that physical exercise can influence learning and memory processes depending on the task and the exercise level (10). There is fair evidence that physicians and therapists should promote physical activity at an intensity level that is adapted to the person’s overall physical capacities as part of a healthy lifestyle for older individuals with and without memory loss (26).

In summary, the present findings indicate that homocysteine administration into the dorsal hippocampus causes learning and memory deficits probably by imbalance in oxidative/antioxidative process and, thus aerobic regular training may reverse this process. However, more studies are obviously needed. Furthermore, another study to establish the effect of stress on learning and memory levels in brain is in progress.

## Authors' Contributions

SH conceived and designed the evaluation, performed parts of laboratory experiments, and the statistical analysis. VDR helped to conceive and design the evaluation, performed parts of laboratory experiments, and interpreted the research results. MP re-evaluated the clinical data, revised the manuscript and drafted the manuscript. All authors read and approved the final manuscript.

## References

[1] Santiard-Baron D, Aupetit J, Janel N (2005). Plasma homocysteine levels are not increased in murine models of Alzheimer’s disease. Neurosci Res.

[2] Cascalheira JF, João SS, Pinhanços SS, Castro R, Palmeira M, Almeida S (2009). Serum homocysteine: Interplay with other circulating and genetic factors in association to Alzheimer's type dementia. ClinBiochem.

[3] Kidd PM (2008). Alzheimer’s disease, amnestic mild cognitive impairment, and age-associated memory impairment: current understanding and progress toward integrative prevention. Altern Med Rev.

[4] Karimi F, Borhani Haghighi A, Petramfar P (2009). Serum levels of homocysteine, vitamin B12, and folic acid in patients with Alzheimer’s disease. Iran J Med Sci.

[5] Zylberstein DE, Lissner L, Björkelund C, Mehlig K, Thelle DS, Gustafson D (2011). Midlife homocysteine and late-life dementia in women. A prospective population study. Neurobiol Aging.

[6] Seshadri S, Beiser A, Selhub J, Jacques PF, Rosenberg IH, D'Agostino RB (2002). Plasma homocysteine as a risk factor for Denentia and Alzheimer s desease. N Engl J Med.

[7] Baydas G, Ozer M, Yasar A, Tuzcu M, Koz ST (2005). Melatonin improves learning and memory performances impaired by hyperhomocysteinemia in rats. Brain Res.

[8] Guilliams TG (2004). Homocysteine – A Risk Factor for Vascular Diseases: Guidelines for the Clinical Practice. J Am Nutraceutl Associ.

[9] Heyn P, Abreu BC, Ottenbacher KJ (2004). The effects of exercise training on elderly persons with cognitive impairment and dementia: a meta-analysis. Arch Phys Med Rehabil.

[10] García-Capdevila S, Portell-Cortés I, Torras-Garcia M, Coll-Andreu M, Costa-Miserachs D (2009). Effects of long-term voluntary exercise on learning and memory processes: dependency of the task and level of exercise. Behavl Brain Res.

[11] Liu-Ambrose T, Donaldson MG (2009). Exercise and cognition in older adults: is there a role for resistance training programmes?. Br J Sports Med.

[12] Naylor AS, Persson AI, Eriksson PS, Jonsdottir IH, Thorlin T (2005). Extended Voluntary Running Inhibits Exercise-Induced Adult Hippocampal Progenitor Proliferation in the Spontaneously Hypertensive Rat. J Neurophysiol.

[13] Cotman WC, Berchtold CN, Christie LA (2007). Exercise builds brain health: key roles of growth factor cascades and inflammation. Trends Neurosc.

[14] Eggermont L, Swaab D, Luiten P, Scherder E (2006). Exercise, cognition and Alzheimer’s disease: More is not necessarily better. Neurosci Biobehav Rev.

[15] Ataie A, Sabetkasaei M, Haghparast A, Hajizadeh Moghaddam A, Ataie R, Nasiraei Moghaddam Sh (2010). An investigation of the neuroprotective effects of Curcumin in a model of Homocysteine - induced oxidative stress in the rat’s brain. Daru.

[16] Van Praag H (2008). Neurogenesis and Exercise: Past and Future Directions. Neuromol Med.

[17] Ravaglia G, Forti P, Maioli F, Chiappelli M, Montesi F, Tumini E (2007). Blood inflammatory markers and risk of dementia: The Conselice Study of Brain Aging. Neurobiol Aging.

[18] Algaidi SA, Christie LA, Jenkinson AM, Whalley L, Riedel G, Platt B (2006). Long-term homocysteine exposure induces alterations in spatial learning, hippocampal signaling and synaptic plasticity. Exp Neurol.

[19] Miller JW (2000). Homocysteine, Alzheimer's disease, and cognitive function. Nutrition.

[20] Jin Y, Brennan L (2008). Effects of homocysteine on metabolic pathways in cultured astrocytes. Neurochem Int.

[21] Van den Kommer TN, Dik MG, Comijs HC, Jonker C, Deeg DJH (2010). Homocysteine and inflammation: Predictors of cognitive decline in older persons? Neurobiol Aging.

[22] Kłapcinska B, Kroemeke A, Tataruch M, Tataruch R, Szołtysek‐Bołdys I, Derejczyk J (2008). Effects of Long‐term Regular Exercise on Cognitive Function, Lipid Profile and Atherogenic Biomarkers in Middle‐aged Men. J Human Kine.

[23] Fabre C, Charmi K, Mucci P, Masse‐Biron J, Prefaut C (2002). Improvement of cognitive function and/or individualized aerobic training in healthy elderly subjects. Int J Sports Med.

[24] Kramer AF, Erickson KI, Colcombe SJ (2006). Exercise, cognition and the aging brain. J Appl Physiol.

[25] Ravaglia G, Forti P, Lucicesare A, Rietti E, Bianchin M, Dalmonte E (2008). Physical activity and dementia risk in the elderly. Neurology.

[26] Massoud F, Belleville S, Bergman H, Kirk J, Chertkow H, Nasreddine Z (2007). Mild cognitive impairment and cognitive impairment, no dementia: Part B, therapy. Alzheimer’s Dementia.

[27] Lange-Asschenfeldt C, Kojda G (2008). Alzheimer’s disease, cerebrovascular dysfunction and the benefits of exercise: From vessels to neurons. Exp Gerontol.

[28] Podewils LJ, Guallar E, Kuller LH, Fried LP, Lopez OL, Carlson M (2005). Physical activity, APOE genotype, and dementia risk: findings from the Cardiovascular Health Cognition Study. Am J Epidemiol.

